# Temporal binding window and sense of agency are related processes modifiable via occipital tACS

**DOI:** 10.1371/journal.pone.0256987

**Published:** 2021-09-10

**Authors:** Agnese Venskus, Francesca Ferri, Daniele Migliorati, Sara Spadone, Marcello Costantini, Gethin Hughes

**Affiliations:** 1 Department of Psychology, University of Essex, Colchester, United Kingdom; 2 Department of Neuroscience, Imaging and Clinical Sciences, University ‘‘G. d’Annunzio”, Chieti, Italy; 3 School of Cognitive Psychotherapy, SPC-APC, Rome, Italy; 4 Department of Psychological, Health and Territorial Sciences, “G. d’Annunzio” University of Chieti-Pescara, Chieti, Italy; 5 Institute for Advanced Biomedical Technologies ‐ ITAB, University “G. d’Annunzio”, Chieti, Chieti, Italy; Psychologische Hochschule Berlin, GERMANY

## Abstract

The temporal binding window refers to the time frame within which temporal grouping of sensory information takes place. Sense of agency is the feeling of being in control of one’s actions, and their associated outcomes. While previous research has shown that temporal cues and multisensory integration play a role in sense of agency, no studies have directly assessed whether individual differences in the temporal binding window and sense of agency are associated. In all three experiments, to assess sense of agency, participants pressed a button triggering, after a varying delay, the appearance of the circle, and reported their sense of agency over the effect. To assess the temporal binding window a simultaneity judgment task (Experiment 1) and a double-flash illusion task (Experiment 2 and 3) was also performed. As expected, the temporal binding window correlated with the sense of agency window. In Experiment 3, these processes were modulated by applying occipital tACS at either 14Hz or 8Hz. We found 14Hz tACS stimulation was associated with narrower temporal biding window and sense of agency window. Our results suggest the temporal binding window and the time window of sense of agency are related. They also point towards a possible underlying neural mechanism (alpha peak frequency) for this association.

## 1 Introduction

Temporal grouping of sensory information is dependent on temporal sensitivity, in other words the ability to detect time-based discrepancy between two stimuli [1]. The time frame within which temporal grouping of sensory information takes place is known as the temporal binding window (TBW). This window is highly variable across individuals [2–4]. Recent reviews [5, 6] of the literature have shown that double-flash illusion is often used to explore the TBW. This task involves simultaneous presentation of visual (flash) and auditory (beep) stimuli followed by the presentation of a second auditory (beep) stimulus after a variable delay. If the second beep occurs within the individuals`TBW then both beeps are integrated with the visual stimulus. This creates an illusion whereby participants report experiencing two flashes despite only one flash being presented. The delay at which an individual no longer perceives two flashes is taken as the width of their TBW, and acts as an index of their temporal sensitivity [5, 6].

There is also evidence suggesting that temporal grouping of sensory information play a pivotal role in sense of agency (SoA) [7–11]. SoA refers to the feeling of being in control of one’s actions and their associated outcomes (i.e. the feeling of having caused a sensory event in the environment). Previous studies have shown that increasing the interval between an action and an associated sensory outcome leads to a reduction in SoA [11]. In addition, SoA seems to depend on temporal grouping of actions and outcomes. For instance, SoA for an outcome can be reduced by the presence of an additional sensory event coinciding with the action because this event, rather than the outcome, is integrated with the action [9]. Farrer and colleagues [8] proposed that if action and outcome occur within a specific temporal interval, within which the action and the outcome are integrated, one experiences a greater SoA. In their study, participants were required to perform an action (a button press) that elicited an outcome (apparition of a circle on a screen) with various delays and to report their sense of agency over that outcome. Findings indicated that participants were more likely to report SoA for the outcome at shorter delays as opposed to longer delays, supporting the notion that SoA depends on the temporal relationship between action and outcome.

Given that temporal grouping of sensory information is an important determinant of SoA, it is plausible that individual differences in TBW are associated with the individual differences in the SoA window. However, existing literature lacks studies exploring this association directly. Therefore, the first aim of the current studies is to investigate the degree to which individual differences in the TBW correlate with individual differences in the SoA window. As both phenomena are linked by temporal grouping of sensory information, a positive correlation would be expected.

If temporal grouping of sensory information links the TBW and SoA, it is plausible that these two processes also have a common underlying neural mechanism. One strong candidate for this would be the frequency of the occipital alpha peak. The power spectrum of human EEG broadly decreases in amplitude as the frequency increases, with some additional peaks at various frequencies, most notably at around 10Hz (see [12] for a comprehensive review). When measured over posterior electrodes, during awake state, this peak is known as the occipital alpha peak. The precise frequency of this peak varies from one person to the next, normally within the range of 8Hz to 12Hz, but can be as low as 7Hz or as high as 14Hz [13, 14].

In terms of the TBW, a higher alpha frequency provides a narrower excitatory phase, and thus results in a higher temporal sensitivity, allowing detection of a shorter temporal discrepancy between two stimuli. As higher temporal sensitivity gives rise to shorter width of the TBW (see [5, 6] for recent reviews), this provides a clear link between alpha peak frequency and the TBW. Studies directly investigating alpha peak frequency and TBW provide further support for this assumption. For example, individual differences in the frequency of the alpha peak have been found to correlate negatively with the width of the TBW [2, 15]. Furthermore, neuromodulation (via tACS) of the frequency of the occipital alpha peak alters the width of the TBW accordingly (increased frequency of the occipital alpha peak is associated with decreased width of the TBW; [2]).

With regards to SoA, the evidence of a possible link to alpha peak frequency is less direct. Nonetheless, as highlighted above, SoA is highly dependent on the interval between action and outcome. Recent evidence has shown that time perception is linked to the TBW [16] and can also be modulated by tACS in the alpha frequency range [13]. These studies suggest that alpha peak frequency may act as a kind of “sample rate” to the visual system, influencing both sensory integration at short intervals (corresponding to an alpha cycle) as well as time perception at longer intervals. As such, if SoA depends in part on the time interval between action and outcome, and on the perception of this interval, then it follows that SoA might also relate not just to the TBW but also to peak alpha frequency. Therefore, the second aim of the current study is to explore whether occipital tACS stimulation alters the width of the SoA window as well as the TBW.

Across three experiments, we first investigated the link between the SoA window, the TBW, and the frequency of the occipital alpha peak. In a first experiment the same participants performed a judgment of agency task [8] and a simultaneity judgment task [17]. The judgment of agency task allowed us to obtain a measure of the SoA window, while the simultaneity judgment task provided a direct measure of the TBW. In a second experiment we used a similar design, but we estimated the TBW indirectly rather than directly (allowing elimination of possible conscious bias present during direct measuring), namely we used the double-flash illusion [2, 18]. In a third experiment we explored whether the tACS stimulation at the upper and lower bounds of the frequency of the occipital alpha peak alters the width of the SoA window similarly to width of the TBW.

## 2 Experiment 1

Experiment 1 investigated the relationship between the SoA window and a direct measure of the TBW. Data is made accessible on a public repository—OSF via the following link https://osf.io/d7rwu/?view_only=89cc3a96ff86437b84874337dea53261.

### 2.1 Method

#### 2.1.1 Participants

The sample consisted of 90 volunteer participants. All participants had normal or corrected to normal vision and hearing to avoid these variables influencing the tasks. Participants were tested at Institute for Advanced Biomedical Technologies in Chieti. The study was approved by the local ethics committee, and participants gave their informed consent before taking part in the study. The study was conducted in accordance with the ethical standards of the 1964 Declaration of Helsinki and approved by the University of G d`Annunzio`s Ethics Committee.

#### 2.1.2 Exclusion criteria

All 90 participants (females = 49, males = 41; mean age = 24.3, SD = 2.6) completed the simultaneity judgment task and judgement of agency task. The data sets that were incomplete were removed from the analysis. Similarly, data sets that did not fit the psychometric sigmoid function (R^2^ less than.4) were removed from further analysis. Six data sets in the simultaneity judgment task and twelve data sets in the judgement of agency task were removed. Hence, the final sample was of 72 participants (females = 40, males = 32; mean age = 24.6, SD = 2.6).

#### 2.1.3 Apparatus/Materials

**Fig 1:**
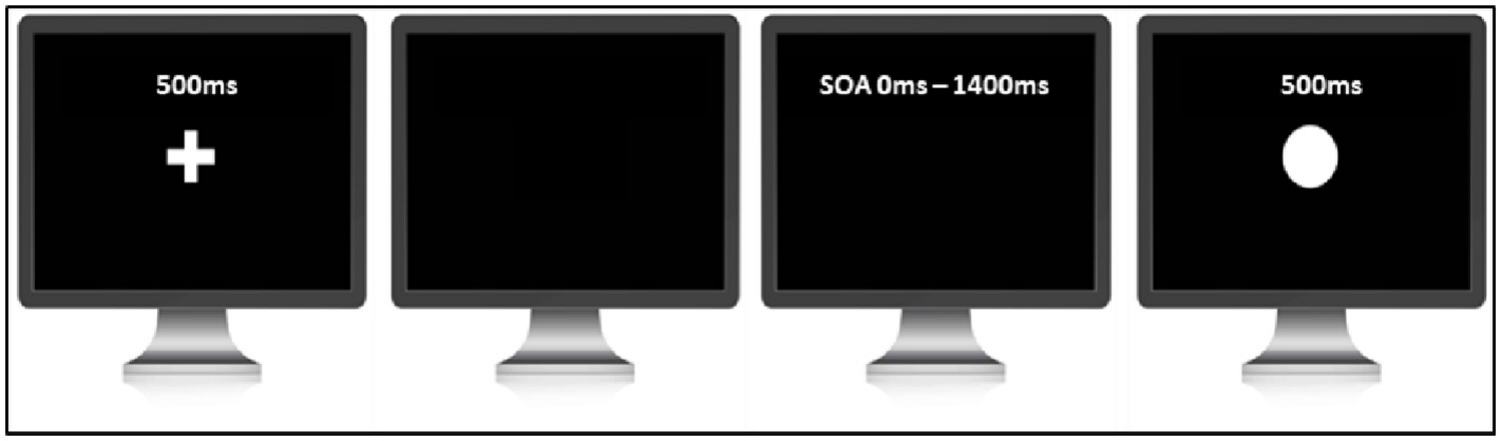
Paradigm of the judgement of agency task.

*2*.*1*.*3*.*1 Judgement of agency task (SoA window measure)*. The judgement of agency task (see Fig 1) was adapted from Farrer and colleagues study [8] All stimuli were presented using MATLAB running Psychtoolbox extension [19, 20] on an LCD monitor with a refresh rate of 60Hz. Each trial started with a white fixation cross in the centre of the monitor. After a delay of 500ms the fixation cross disappeared, signalling the beginning of the trial. After the cross disappeared, participants were asked to press the space bar on the computer keyboard whenever they wanted. Once participants pressed the key, a grey circle of 2.5cm in diameter was displayed in the centre of the screen for 500ms with 11 possible delays ranging from 0ms to 1400ms in steps of 140ms. The task consisted of 2 blocks with each delay being presented 10 times in random order. In total participants completed 220 trials. Participants were required to judge if the appearance of the circle was caused by their button press, or if the computer had triggered the circle to appear. Participants were told that on some trials the computer would cancel their button press and re-trigger the appearance of the circle at a random interval. Participants needed to press key `1`if they thought that it was most likely they triggered the circle to appear and `2`if they thought that it was most likely computer triggered the circle to appear. This response approach was chosen over that of that of Farrer and colleagues [8], where participants were given three choices (i.e. full control, partial control and no control), to avoid participants opting for the partial control if not fully sure. Such partial control responses would complicate the calculation of the time window of SoA, via the fitting of a sigmoid function.

**Fig 2:**
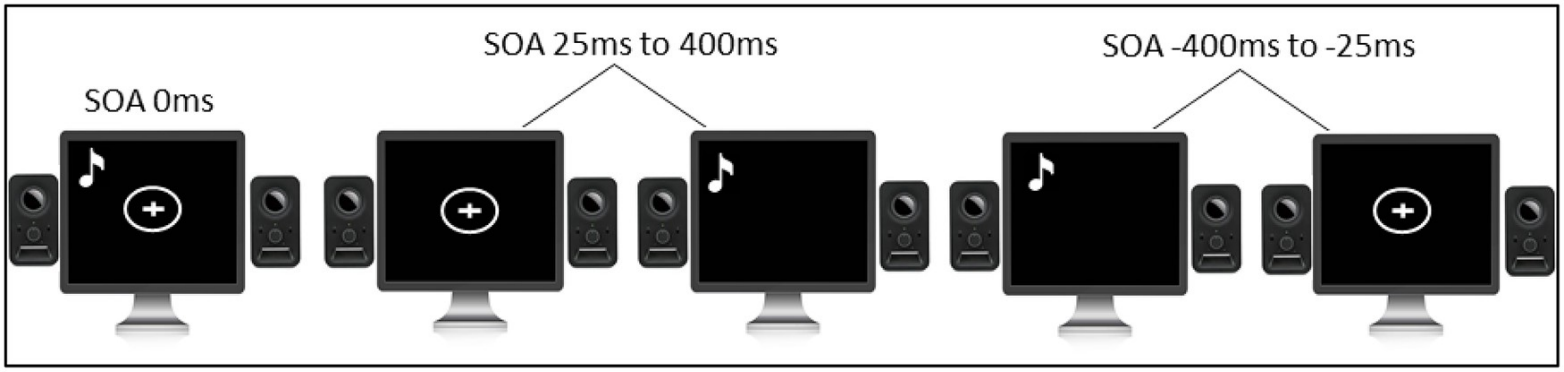
Paradigm of the simultaneity judgement task.

*2*.*1*.*3*.*2 Simultaneity Judgment Task (TBW measure)*. All stimuli were presented using MATLAB running the Psychtoolbox extension [19, 20] on an LCD monitor with a refresh rate of 60Hz (see Fig 2). Visual stimuli consisted of a white ring circumscribing a visual fixation cross in the middle of the monitor on a black background. Visual stimuli were 1.8cm in diameter and lasted 33.3ms. Auditory stimuli consisted of a 3500Hz pure tone and lasted for 33.3ms. They were presented via two typical PC stereo speakers. In order to minimise the possible effect of the spatial cues on the perception of the task speakers were placed at each side of the monitor and raised to align with the position of the visual stimuli [21]. Visual and auditory stimuli were delivered simultaneously or non-simultaneously with one of the following Stimulus Onset Asynchronies: 0ms, ±25ms, ±50ms, ±75ms, ±100ms, ±150ms, ±200ms, ±300ms, ±400ms. Negative Stimulus Onset Asynchronies indicate trials in which the auditory stimulus was presented first (auditory leading trials), while positive Stimulus Onset Asynchronies indicate trials in which the visual stimulus was presented first (visual leading trials). Participants performed two blocks, with a 5mins break between the blocks. In each block, each Stimulus Onset Asynchrony was presented 12 times for a total of 204 trials per block, in a random order. Overall participants completed 408 trials. Participants were instructed to report whether the auditory and visual stimuli were presented at the same time or different times by pressing the key `l`or the key `s`respectively.

#### 2.1.4 Procedure

Participants were seated in a dimly lit room approximately 60cm away from the computer screen with the plane of their eyes aligned to the centre of the monitor. Participants completed the simultaneity judgment task and the judgement of agency task. The order of the tasks was counterbalanced across participants.

#### 2.1.5 Data analysis

To examine the SoA window, the number of times the individual reported that they caused the circle to appear on the screen was recorded. Thereafter, a sigmoid function was fitted to the data, determining each participant`s inflection point (corresponding to the width of the SoA window), in ms. A decreasing sigmoid function was used to fit the distribution of responses and was defined by the equation: y = a+b/(1+exp(-(x-c)/d)) (a = upper asymptote; b = lower asymptote; c = inflection point; d = slope). For each participant, c was taken as the SoA window, i.e. the point of decay of the self-attribution [22, 23]. To explore the TBW, first the percentage of simultaneous responses across all Stimulus Onset Asynchronies for each participant was computed. The observed distribution of responses was fitted to a Gaussian function using the fit function implemented in MATLAB (a1*exp(-((x-b1)/c1)^2). The average interval of visual-first and auditory-first values at which participants responded with 75% synchronous responses was taken as a measure of the width of the TBW. The above method of analysis was chosen as the Gaussian fitting is the standard in this kind of analysis, as well as 75% response criterion [24, 25]. To investigate the relationship between TBW and SoA window, we ran a correlation analysis between individuals`TBW and SoA window.

### 2.2 Results

#### 2.2.1 Relationship between TBW and SoA window

**Fig 3:**
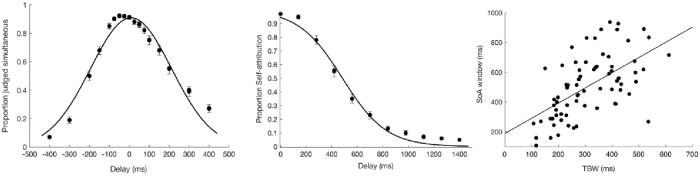
Relationship between TBW and SoA window. (A) Across-participants average probability of judging the stimuli as simultaneous plotted as a function of the delay. The curve represents the Gaussian fit determining the amplitude of TBW, corresponding to the standard deviation of the Gaussian curve. The points represent across-participants average raw data with error bars showing standard error of the mean. (B) Across-participants average probability of self-attribution plotted as a function of the delay. The curve represents the sigmoid fit determining the amplitude of the SoA window corresponding to the inflection point of the sigmoid. The points represent across-participants average raw data with error bars showing standard error of the mean. (C) Linear correlation between TBW (simultaneity judgment task) and the SoA window (judgment of agency task).

A Pearson correlation indicated that there was a moderate to strong positive relationship between the width of the TBW (M = 327ms, SD = 75ms) and SoA window (M = 551ms, SD = 203ms), r(70) = .59, p < 0.001 (see Fig 3).

## 3 Experiment 2

Experiment 2 investigated relationship between SoA window and an indirect measure of the TBW. Data is made accessible on a public repository—OSF via the following link https://osf.io/d7rwu/?view_only=89cc3a96ff86437b84874337dea53261.

### 3.1 Method

#### 3.1.1 Participants

The sample consisted of 49 volunteer participants. All participants had normal or corrected to normal vision and hearing to avoid these variables influencing the tasks. Participants were tested at Institute for Advanced Biomedical Technologies in Chieti. The study was approved by the local ethics committee, and participants gave their informed consent before taking part in the study. The study was conducted in accordance with the ethical standards of the 1964 Declaration of Helsinki and approved by the University of G d`Annunzio`s Ethics Committee.

#### 3.1.2 Exclusion criteria

All 49 participants (females = 29, males = 20; mean age = 24.5, SD = 2.9) completed the double-flash illusion and judgement of agency task. The data sets that were incomplete or showed failure to perceive the double-flash illusion task (not experiencing the illusion on any of the trials) were removed from further analysis. Also data sets that did not fit the psychometric sigmoid function (R^2^ less than.4) were removed from further analysis. Fifteen data sets were excluded. Hence, the final sample was of 34 participants (females = 20, males = 14; mean age = 24, SD = 2.5).

#### 3.1.3 Apparatus/Materials

*3*.*1*.*3*.*1 Judgement of agency task (SoA window measure)*. The SoA task was the same as in Experiment 1.

**Fig 4:**
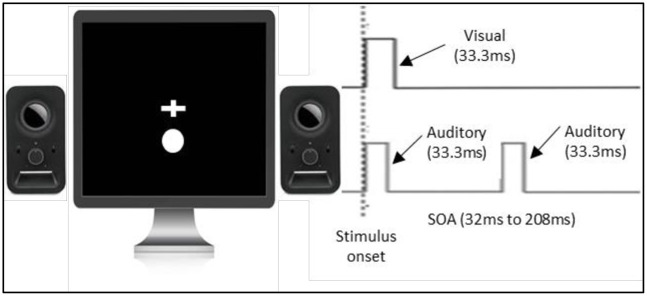
Paradigm of the double-flash illusion task.

*3*.*1*.*3*.*2 Double-flash illusion (TBW measure)*. All stimuli were presented using MATLAB running Psychtoolbox extension [19, 20] on the LCD monitor with a refresh rate of 60Hz (see Fig 4). Visual stimuli consisted of a white circle 1.32cm in diameter and lasted for 33.3ms. Visual stimuli were located 1 cm below the fixation cross that was positioned in the centre of the screen, as placing the stimuli in peripheral vision allows for better illusionary percept [26]. Auditory stimuli consisted of a 3500Hz pure tone and lasted for 33.3ms. They were presented via two typical PC stereo speakers. In order to minimise the possible effect of the spatial cues on the perception of the task, speakers were placed at each side of the monitor and raised to align with the position of the visual stimuli [21]. Each trial started with a white fixation cross in the centre of the monitor that remained on the screen throughout the trial. On each trial, visual and auditory stimuli were presented simultaneously, and after a variable Stimulus Onset Asynchrony (randomly chosen between 32ms and 208ms, in steps of 16ms) a second auditory stimulus was presented. These particular Stimulus Onset Asynchronies were chosen to synchronize the stimulus timing with the refresh rate of the screen (60Hz, 16.6ms). Participants performed one block. Each Stimulus Onset Asynchrony was presented 18 times, for a total 216 trials. Participants were instructed to fixate on the fixation cross and report whether they perceived one or two flashes by pressing the key `l`or the key `s`respectively.

#### 3.1.4 Procedure

Participants were seated in a dimly lit room approximately 60cm away from the computer screen with the plane of their eyes aligned to the centre of the monitor. Participants completed double-flash illusion and judgement of agency task. The order of the two tasks was counterbalanced across participants.

#### 3.1.5 Data analysis

Data for the judgement of agency task were treated in the same way as in Experiment 1. To assess the width of the TBW, the time window in which the illusion was maximally perceived, the percentage of trials where two flashes were reported was first plotted as a function of the inter-beep delay. A psychometric sigmoid function was then fitted to the data. The sigmoid function was defined by the equation: y = a+b/(1+exp(-(x-c)/d)) (a = upper asymptote; b = lower asymptote; c = inflection point; d = slope). For each participant, c was taken as the TBW, i.e. the point of decay of the illusion [22, 23]. To investigate the relationship between the indirect measure of the TBW and the SoA window we ran a correlational analysis between the two variables.

### 3.2 Results

#### 3.2.1 Relationship between TBW and SoA window

**Fig 5:**
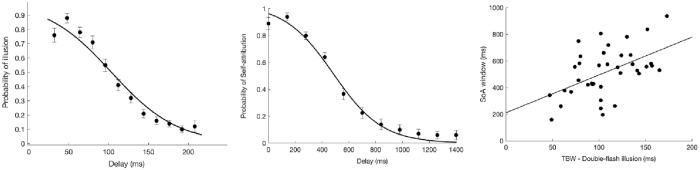
Relationship between TBW and SoA window. (A) Across-participants average probability of perceiving the illusion plotted as a function of inter-beep delay. The curve represents the sigmoid fit determining the amplitude of the window of illusion, corresponding to the inflection point of the sigmoid. The points represent across-participants average raw data with error bars showing standard error of the mean. (B) Across-participants average probability of self-attribution plotted as a function of the delay. The curve represents the sigmoid fit determining the amplitude of the SoA window corresponding to the inflection point of the sigmoid. The points represent across-participants average raw data with error bars showing standard error of the mean. (C) Linear correlation between TBW (double-flash illusion) and the SoA window (judgement of agency task).

A Pearson correlation between the TBW (M = 102ms, SD = 20) and the SoA (M = 580ms, SD = 180ms) window showed moderate positive relationship, r(32) = .39, p = 0.002 (see Fig 5).

## 4 Experiment 3

Experiment 3 aimed to explore whether the tACS stimulation at the higher or lower bounds of the frequency of the occipital alpha peak alters the width of the time window of SoA. It also aimed to replicate previous findings showing that the TBW can be modulated by occipital tACS stimulation. The study was preregistered on AsPredicted, accessible via https://aspredicted.org/dh38n.pdf. Data is made accessible on OSF via the following link https://osf.io/d7rwu/?view_only=89cc3a96ff86437b84874337dea53261.

### 4.1 Method

#### 4.1.1 Participants

The sample consisted of 45 participants, as per the preregistration. Participants consisted of student volunteers from the University of Essex recruited via the University research advertisement websites. All participants had normal or corrected to normal vision and hearing to avoid these variables influencing the tasks. The local ethics committee approved the study, and participants gave their informed consent before taking part in the study. The study was conducted in accordance with the ethical standards of the 1964 Declaration of Helsinki and approved by the University of Essex`s Faculty Ethics Subcommittee (departmental reference no: AV1901).

#### 4.1.2 Exclusion criteria

In total 45 participants took part in the study (females = 35, males = 10; mean age = 21.8, SD = 5.9). The data of those participants that failed to complete the entire study or failed to perceive the double-flash illusion task (not experiencing the illusion on any of the trials) was removed from further analysis. Similarly, as per the preregistration, those data sets that did not fit the psychometric sigmoid function (R^2^ less than.4) were removed from further analysis. After exclusion, the SoA task included a final sample of 33 data sets (females = 26, males = 7; mean age = 22.2, SD = 6.4), while the TBW task included a final sample of 32 data sets (females = 25, males = 7; mean age = 20.7, SD = 3.4).

#### 4.1.3 Design

The current study used a within-subjects design. The independent variable in the study was the tACS stimulation at 2 frequencies (8Hz and 14Hz). All participants experienced both frequencies of the tACS stimulation. The order of the tACS sessions was counterbalanced, such that half of the participants completed the 8Hz condition first while the other half completed 14Hz condition first. The dependent variables were the width of the TBW and the width of the SoA window measured at both frequencies.

#### 4.1.4 Apparatus/Materials

*4*.*1*.*4*.*1 tACS stimulation*. tACS was delivered by a battery-powered DC stimulator (Magstim, UK) via two rubber electrodes enclosed in saline-soaked sponges. Sponges containing the electrodes were imbedded into an EEG cap to keep the electrodes securely attached on the head. The reference electrode was located on the vertex (Cz), whilst the stimulation electrode was located on the occipital cortex (Oz). In order to decrease the current density at the reference location, the reference electrode was 35cm^2^ whereas stimulation electrode was 9cm^2^. A sinusoidal waveform current was used, the DC offset was set at 0, the intensity of the stimulation was set at 2 mA peak to peak (10 seconds fade in) and the impedance was set below 5k. The above protocol is a replication of the protocol employed by Cecere and colleagues [2].

**Fig 6:**
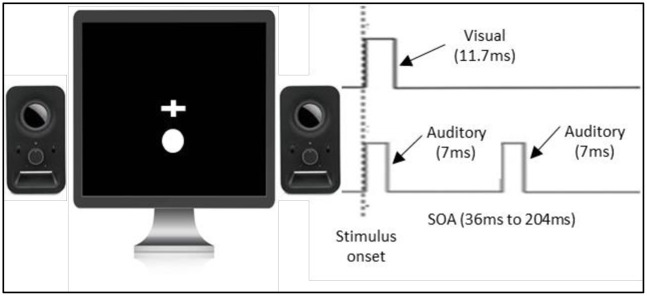
Paradigm of the double-flash illusion task.

*4*.*1*.*4*.*2 Double-flash illusion (TBW measure)*. The double-flash illusion used was the same as that in the study of Cecere and colleagues [2] (see Fig 6). We chose to use this version of the task, as it has previously been associated with individual differences in the EEG alpha peak frequency, and has also been successfully modulated by tACS [2]. The basic premise of the task is the same as that used in Experiment 2, with some small differences. Notably, auditory stimuli were presented for 7ms and visual stimuli for 11.7ms instead of 33.3ms as the above stimuli duration has been demonstrated to allow the multisensory integration to be tested accurately [2–4]. The inter-beep intervals were taken from Cecere and colleagues study [2] and consisted of 300 trials with each possible inter-beep interval presented 20 times. Inter-beep intervals ranged from 36ms to 204ms in 12ms steps. The above range of inter-beep intervals was chosen as Cecere and colleagues`study [2] showed that such methodology not only captures but also extends beyond the time frame within which the double-flash illusion task is perceived in the general population. All stimuli were presented on a CRT monitor with a refresh rate of 85Hz.

*4*.*1*.*4*.*3 Judgement of agency task (SoA window measure)*. The SoA task was the same as in Experiment 1 and 2.

#### 4.1.5 Procedure

Before undertaking the study tACS intensity was adjusted to each participant individually. Namely, tACS intensity was initially set to 2000 μA reduced until the participant no longer experiences phosphenes. Thresholds ranged from 1600 μA to 2000 μA. Participants were seated in a dimly lit room approximately 60cm away from the computer screen with the plane of their eyes aligned to the centre of the monitor. Participants completed the double-flash illusion and the judgement of agency task while receiving continuous tACS at 8Hz or 14Hz. Approximately a week (7 +/- 1 day) after the initial tasks participants completed the same two tasks while receiving continuous tACS at 8Hz or 14Hz depending which frequency of occipital alpha cycle was used previously. The order of the tasks was counterbalanced across participants.

#### 4.1.6 Data analysis

The SoA window and the TBW were calculated as in Experiment 2 and preregistered on AsPredicted (https://aspredicted.org/dh38n.pdf).

### 4.2 Results

#### 4.2.1 Effect of occipital tACS stimulation on TBW and SoA window

**Fig 7:**
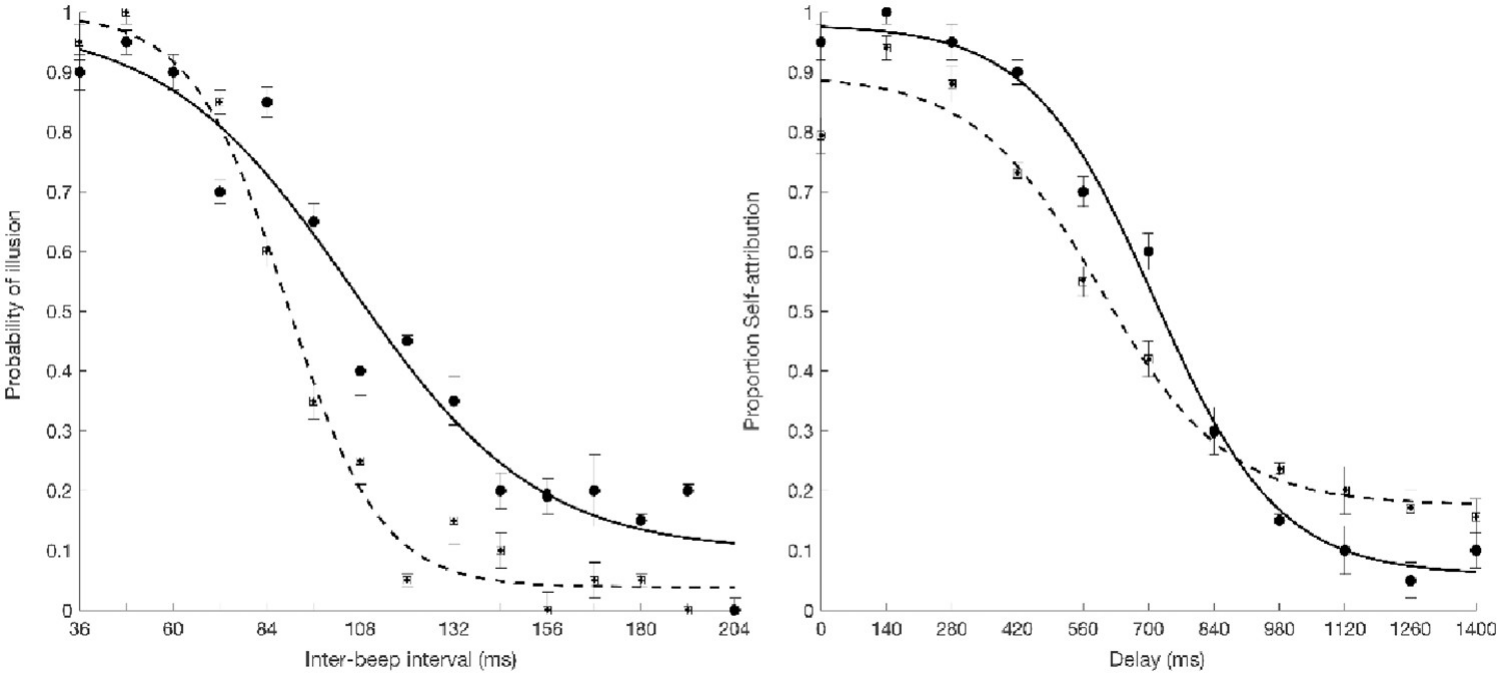
Effect of occipital tACS stimulation on TBW and SoA window. (A) Average percentage of probability of perceiving the illusion plotted as a function of inter-beep delay. A sigmoid function was then fitted to the averaged data points. The black solid curve represents the sigmoid fit determining the point of decay of the illusion, corresponding to the width of the TBW, in 8Hz condition. The black dotted curve represents the sigmoid fit determining the point of decay of the illusion, corresponding to the width of the TBW, in 14Hz condition. The points represent across-participants average raw data with error bars showing standard error of the mean. (B) Average percentage of probability of self-attribution plotted as a function of delay. Data of each participant was first averaged for each delay and hereafter across all participants according to the delays. A sigmoid function was then fitted to the averaged data points. The black solid curve represents the sigmoid fit determining the point when the SoA judgement changes, corresponding to the width of the SoA window, in 8Hz condition. The black dotted curve represents the sigmoid fit determining the point when the SoA judgement changes, corresponding to the width of the SoA window, in 14Hz condition. The points represent across-participants average raw data with error bars showing standard error of the mean.

By examining the group averaged raw data and the sigmoid curve it appears that the width of the TBW and the width of the SoA window is wider at 8Hz condition than 14Hz condition (see Fig 7).

A paired-samples t-test confirmed that the width of the TBW was wider at 8Hz (M = 108ms, SD = 25ms) than 14Hz (M = 91ms, SD = 28ms), t(31) = 3.2, p = .003, d = 0.64, observed power = 0.88. Similarly, a paired-samples t-test showed that the width of SoA window was wider at 8Hz (M = 700ms, SD = 266ms) than 14Hz (M = 605ms, SD = 268ms), t(32) = 2.2, p = .034, d = 0.36, observed power = 0.58. Taken together these findings suggest that tACS stimulation at the upper or lower bounds of the frequency of the occipital alpha peak, alters the width of the SoA window similarly to width of the TBW.

#### 4.2.2 Relationship between TBW and SoA window

To test whether the SoA window is related to the TBW a correlation between the above variables was conducted. We conducted separate correlations within each stimulation session to assess the relationship between the SoA window and the TBW. Results of the Pearson correlation indicated that there was no relationship between the width of the TBW and SoA window in 8Hz condition, r(28) = -.061, p = .758, or in the 14Hz condition, r(28) = .291, p = .133.

## 5 Discussion

One of the aims of the current study was to explore the relationship between the TBW and SoA. As both phenomena are linked by temporal grouping of sensory information, it was predicted that a positive correlation will be found. Another aim was to explore whether the tACS stimulation at the upper and lower bounds of the frequency of the occipital alpha peak alters the width of the SoA window and TBW.

### 5.1 Relationship between TBW and SoA window

With respect to the direct relationship between the TBW and SoA window, this study was the first to directly address this question. While the first two experiments provide support for the link between these two processes, this relationship was not observed in the final experiment. How might we understand this inconsistent pattern of results? Although an important aspect of both TBW and SoA window is the temporal grouping of sensory information, this connection is stronger for the TBW compared to SoA window. Indeed, SoA is known to be modulated by many different cues [27, 28], whereas TBW is purely a measure of temporal sensitivity. More specifically, in addition to the temporal relationship between action and outcome, SoA can also be influenced by action selection fluency [27, 29] as well as the consistency between the predicted and the actual outcome. The former refers to a notion that sense of agency is influenced by the action selection in advance of the action itself, and before action outcomes are known [30]. In the latter case, this can be explained both in terms of predictive processes [31], and in terms of postdictive confabulation [32]. This calculation might also include integrating prior information to inform our SoA [28]. Such prior information may include (but might not be limited to) possible alternative causes of the observed action outcome, or whether or not the action was freely selected. As such, although SoA is likely informed by cues related to temporal integration, and therefore will relate to the TBW, this relationship will inevitably be incomplete. Hence, although linked, SoA and TBW are also partially dissociable.

Given the fact that SoA depends on many different cues, one might also expect this to vary in different contexts, and also between individuals [33]. In latter case for instance, some participants might rely more on temporal cues, and less on action-related, or other cues. With respect to different contexts, we must mention that one important difference in Experiment 3 is that participants were undergoing continuous tACS stimulation while completing the judgement of agency task. As such, it is possible that the presence of tACS stimulation may have been responsible for removing the association between TBW and SoA window. This could occur either directly, as a result of stimulating the brain, or indirectly, as a result of the change in context. Indeed, previous research has shown that electrical brain stimulation can alter the degree to which participants integrate information about action-outcome congruency when generating judgements of agency [34]. Indirect effects might be less prevalent in the double-flash illusion task, which predominately utilises temporal cues. Further research should investigate this possible explanation in more detail, for instance by including additional baseline conditions and/or sham stimulation conditions.

It is also worth noting that Experiment 3 used a slightly different version of the double-flash illusion task. We chose this version of the task as it has previously been successfully modulated by tACS [2]. Although the different tasks we used to measure TBW share many common features and are grounded in the same theoretical framework, subtle differences between the tasks may lead to slight differences in the degree to which they correlate with the SoA window. Hence, further research should also explore the possibility that the relationship between TBW and SoA window vary somewhat dependent on the precise task features. Nonetheless, the association observed between these two processes in Experiment 1 and 2, using very different TBW tasks, suggests that task specific factors may be less important than other factors such as context.

We should additionally note some limitations with the behavioural tasks employed in the current studies. In contrast to Farrer and colleagues [8], we opted to only allow participants to respond that either they had caused the circle to appear, or that the computer had caused the circle to appear. This was in contrast to the original study, where participants could also report partial control. We made this change to allow simpler calculation of the SoA window, and note that in Farrer and colleagues study [8] participants did report no-control in the majority of trials at the longest delays. Nonetheless, one previous study [7] reported that a temporal delay was only sufficient to introduce uncertainty but not completely remove the SoA. That study differed to the method used here though, as participants made continuous joystick movements. One additional limitation of the judgement of agency task in the current studies is that despite participants being told that the computer would sometimes cancel their button press and take over control of the appearance of the circle, every trial did include both a button press and the appearance of the circle. This means that participants might not have fully believed that they were not ultimately in control of the appearance of the circle. Future studies should investigate these issues in more detail. For instance, it would be interesting to examine the relationship between the SoA window and temporal sensitivity when participants can also report partial-control and when agency is manipulated in more complex ways, such as by changing the contingencies between the action and the sensory event. Finally, with respect to the double-flash illusion task, we decided not to include trials where participants were actually presented no flashes or two flashes, to control for response biases [6]. We note that our task is consistent with previous studies on which this study was based [2, 3]. We also note that no participants reported being suspicious about the fact that only one flash was ever presented. Nonetheless, future studies should attempt to overcome these possible response biases by including these additional trials.

### 5.2 Modulation of TBW and SoA window by occipital tACS

In terms of the second aim of the current study, we found the occipital tACS stimulation at the upper bound of the alpha frequency range (14Hz), reduced the width of both the TBW and the SoA window, compared to stimulation at the lower bound of the alpha range (8Hz). These results support previous findings in the field [2, 15, 35], which have linked the alpha peak frequency to the width of the TBW. Moreover, the current study revealed novel evidence suggesting that alpha peak frequency is also related to the SoA window. As discussed earlier, this association might be explained by the observation that tACS has also been shown to influence time perception [13], which in turn will feed into the time window of agency.

However, it must be noted that as we did not employ a tACS condition at any other electrode locations (a control condition), it is not possible to rule out the possibility that the tACS modulation was not directly related to the occipital alpha cycle. For instance, stimulation of the peripheral nerves could cause tactile sensation, which in turn could then drive the effect of the stimulation as opposed to the frequency of the alpha peak. However, this possibility seems to be unlikely. Firstly, participants were actively encouraged to report any scalp tactile sensation not only during the set-up but throughout the experiment. Debrief confirmed that none of the participants experienced scalp tactile sensation once the experiment began. Hence, any tactile sensation would be below perceptual threshold and hence unlikely to induce the effect of the stimulation. It is worth noting that the previous studies in this field on which this research was based [2, 13], also did not include any additional control montage. Nevertheless, to ensure that sub threshold tactile sensation is not interfering with the findings (in this and other studies), further research should add a control condition (using different montage i.e. stimulation excluding modulation of the frequency of the occipital alpha peak) to rule out more generalised effects of tACS simulation.

## 6 Conclusions

Firstly, the current study explored the relationship between the TBW and SoA. In the first two experiments, we found a consistent and reliable association between an individual’s TBW and their SoA window, while the final experiment did not show this relationship. In the final experiment we showed that both the TBW and the SoA window is modifiable via tACS stimulation of the upper versus lower bound of the frequency of the alpha peak. This provides novel evidence for a possible common neural mechanism linking the TBW and the time window of SoA, namely the frequency of the occipital alpha peak.
